# Supplementary material for: Genetic diversity and demographic history of the leopard seal: A Southern Ocean top predator
Source: PLoS One. 2023 Aug 11;18(8):e0284640. doi: 10.1371/journal.pone.0284640 (PMC10420386; doi:10.1371/journal.pone.0284640)
Supplement: S1 File — All individuals were tagged and sampled at Cape Shirreff, Livingston Island by the U.S. Antarctic Marine Living Resources (U.S. AMLR). GenBank accession numbers OQ451774 — OQ451802 correspond to new haplotypes detected in this study. (DOCX) [file pone.0284640.s001.docx]

**Supporting Information**

**S1 Table.** Leopard seals, *Hydrurga leptonyx*, sequenced for mtDNA control region (405bp). All individuals were tagged and sampled at Cape Shirreff, Livingston Island by the U.S. Antarctic Marine Living Resources (AMLR). GenBank accession numbers OQ451774 ⎯ OQ451802 correspond to new haplotypes detected in this study*.

| U.S. AMLR Field Tag # | Field  Season | Sex | GenBank accession # | Haplotype  Source |
| --- | --- | --- | --- | --- |
| 115266^a^ | 2011-2012 | NA | OQ451774 | This study |
| 403 | 2009-2010 | Female | OQ451774 | This study |
| 401 | 2009-2010 | Female | OQ451774 | This study |
| 36 | 2011-2012 | Female | OQ451774 | This study |
| 148 | 2018-2019 | Female | OQ451774 | This study |
| 26 | 2011-2012 | Female | OQ451774 | This study |
| 42 | 2011-2012 | Female | OQ451774 | This study |
| 16 | 2013-2014 | Female | OQ451774 | This study |
| 88 | 2014-2015 | Female | OQ451774 | This study |
| 39 | 2011-2012 | NA | MW168809 | Hernández-Ardila et al., 2021 |
| 405 | 2009-2010 | Female | MW168809 | Hernández-Ardila et al., 2021 |
| 133 | 2017-2018 | Female | OQ451775 | This study |
| 111 | 2016-2017 | Male | OQ451775 | This study |
| 48 | 2011-2012 | Female | OQ451775 | This study |
| 84 | 2013-2014 | Female | OQ451775 | This study |
| 82 | 2016-2017 | Male | MW168807 | Hernández-Ardila et al., 2021 |
| 10 | 2008-2009 | Male | MW168807 | Hernández-Ardila et al., 2021 |
| 72 | 2012-2013 | Female | MW168807 | Hernández-Ardila et al., 2021 |
| 149 | 2018-2019 | NA | MW168807 | Hernández-Ardila et al., 2021 |
| 129 | 2017-2018 | Male | MW168807 | Hernández-Ardila et al., 2021 |
| 13 | 2008-2009 | Female | MW168807 | Hernández-Ardila et al., 2021 |
| 52 | 2011-2012 | Female | MW168807 | Hernández-Ardila et al., 2021 |
| 6 | 2008-2009 | Female | OQ451776 | This study |
| 8 | 2008-2009 | Female | OQ451776 | This study |
| 119 | 2016-2017 | Male | OQ451776 | This study |
| 139 | 2017-2018 | Female | OQ451777 | This study |
| 145 | 2017-2018 | Female | OQ451777 | This study |
| 43 | 2011-2012 | Female | OQ451777 | This study |
| 87 | 2013-2014 | Female | OQ451777 | This study |
| 402 | 2009-2010 | Female | MW168806 | Hernández-Ardila et al., 2021 |
| 60 | 2012-2013 | Female | OQ451778 | This study |
| 74 | 2014-2015 | Female | OQ451779 | This study |
| 124 | 2017-2018 | Female | OQ451779 | This study |
| 406 | 2008-2009 | Female | OQ451779 | This study |
| 394(G) | 2008-2009 | Female | OQ451779 | This study |
| 120 | 2013-2014 | Male | OQ451779 | This study |
| 394(Y) | 2008-2009 | Female | OQ451780 | This study |
| 398 | 2008-2009 | Female | OQ451780 | This study |
| 137 | 2017-2018 | Female | OQ451781 | This study |
| 135 | 2017-2018 | Female | OQ451782 | This study |
| 34 | 2011-2012 | Female | OQ451783 | This study |
| 142 | 2017-2018 | Female | OQ451784 | This study |
| 422 | 2008-2009 | Female | OQ451784 | This study |
| 146 | 2018-2019 | Female | OQ451785 | This study |
| 127 | 2017-2018 | Female | OQ451785 | This study |
| 78 | 2013-2014 | Male | OQ451785 | This study |
| 397 | 2013-2014 | Female | OQ451785 | This study |
| 80 | 2013-2014 | Female | OQ451785 | This study |
| 20 | 2009-2010 | Female | OQ451786 | This study |
| 14 | 2008-2009 | Male | OQ451787 | This study |
| 141 | 2017-2018 | Male | OQ451788 | This study |
| 46 | 2011-2012 | Female | OQ451789 | This study |
| 107 | 2016-2017 | Female | MW168805 | Hernández-Ardila et al., 2021 |
| 0 | 2009-2010 | Female | MW168805 | Hernández-Ardila et al., 2021 |
| 128 | 2017-2018 | Female | MW168805 | Hernández-Ardila et al., 2021 |
| 9 | 2008-2009 | Female | MW168805 | Hernández-Ardila et al., 2021 |
| 411 | 2009-2010 | Female | MW168805 | Hernández-Ardila et al., 2021 |
| 99 | 2014-2015 | Female | MW168805 | Hernández-Ardila et al., 2021 |
| 11 | 2014-2015 | Female | MW168805 | Hernández-Ardila et al., 2021 |
| 138 | 2017-2018 | Female | OQ451790 | This study |
| 118 | 2016-2017 | Male | OQ451791 | This study |
| 22 | 2009-2010 | Female | OQ451792 | This study |
| 102 | 2014-2015 | Female | OQ451792 | This study |
| 100 | 2017-2018 | Female | OQ451792 | This study |
| 17 | 2009-2010 | Female | OQ451793 | This study |
| 41 | 2011-2012 | Female | OQ451794 | This study |
| 89 | 2014-2015 | NA | OQ451795 | This study |
| 108 | 2016-2017 | Female | OQ451796 | This study |
| 71 | 2013-2014 | Female | OQ451796 | This study |
| 96 | 2016-2017 | NA | OQ451797 | This study |
| 140 | 2017-2018 | Male | OQ451798 | This study |
| 67 | 2012-2013 | NA | MW168802 | Hernández-Ardila et al., 2021 |
| 63 | 2013-2014 | Female | MW168802 | Hernández-Ardila et al., 2021 |
| 143 | 2017-2018 | Female | MW168802 | Hernández-Ardila et al., 2021 |
| 144 | 2017-2018 | Male | MW168802 | Hernández-Ardila et al., 2021 |
| 152 | 2018-2019 | Female | MW168802 | Hernández-Ardila et al., 2021 |
| 70 | 2009-2010 | Female | MW168802 | Hernández-Ardila et al., 2021 |
| 58 | 2012-2013 | Female | MW168802 | Hernández-Ardila et al., 2021 |
| 81 | 2012-2013 | Female | MW168802 | Hernández-Ardila et al., 2021 |
| 37 | 2013-2014 | Female | MW168802 | Hernández-Ardila et al., 2021 |
| 57 | 2017-2018 | Female | MW168802 | Hernández-Ardila et al., 2021 |
| 112 | 2016-2017 | Female | OQ451799 | This study |
| 85 | 2013-2014 | Female | OQ451799 | This study |
| 377 | 2009-2010 | Female | OQ451800 | This study |
| 59 | 2012-2013 | Female | OQ451800 | This study |
| 77 | 2013-2014 | Male | OQ451800 | This study |
| 18 | 2009-2010 | Female | OQ451801 | This study |
| 395 | 2008-2009 | Female | OQ451801 | This study |
| 132 | 2017-2018 | Female | OQ451802 | This study |
| 45 | 2011-2012 | Female | OQ451802 | This study |

^a^Untagged seal. Number corresponds to the Marine Mammal and Sea Turtle Collection Research Tissue Collection number (LABID) at the NOAA Southwest Fisheries Science Center, La Jolla, CA.

* We compared 34 haplotypes detected in our study to leopard seal mtDNA control region sequences available in GenBank (accession # MW168801—MW168812 (Hernández-Ardila et al., 2021); U03590.1 (Slade et al., 1994); AM181026 (Arnason et al., 2006)). All sequences were trimmed in order to produce an alignment (402bp). Three pre-existing sequences were eliminated from the alignment due to length issues (MW168801; MW168803; MW168804). As a result, we detected five haplotypes in our dataset reported by Hernández-Ardila et al. (2021); 29 were had not been previously identified.

**References**

Arnason U, Gullberg A, Janke A, Kullberg M, Lehman N, Petrov EA, Väinölä R. (2006) Pinniped phylogeny and a new hypothesis for their origin and dispersal. Mol Phylogenet Evol. 41(2):345-54. doi: 10.1016/j.ympev.2006.05.022.

Hernández-Ardila, L.V., Barragán-Barrera, D.C., Negrete, J., Poljak, S., Riet-Sapriza, F.G., and Caballero, S. (2021). Insights into the genetic diversity of the leopard seal (*Hydrurga leptonyx*), inferred from mitochondrial DNA analysis, at Danco Coast, Antarctic Peninsula. Boletín de Investigaciones Marinas y Costeras-INVEMAR 50, 227-238.

Slade RW, Moritz C, Heideman A. (1994) Multiple nuclear-gene phylogenies: application to pinnipeds and comparison with a mitochondrial DNA gene phylogeny. Mol Biol Evol. 341-56. doi: 10.1093/oxfordjournals.molbev.a040117.
